# DLFE-Net: Preserving Details and Removing Noise Using HVI Color Space for Low-Light Image Enhancement

**DOI:** 10.3390/s25175353

**Published:** 2025-08-29

**Authors:** Zhaokun He, Xin Yuan, Guozhu Hao, Wei Wang

**Affiliations:** 1 School of Computer Science and Technology, Wuhan University of Science and Technology, Wuhan 430065, China; hezhaokun0602@163.com (Z.H.); wangwei8@wust.edu.cn (W.W.); 2 Hubei Province Key Laboratory of Intelligent Information Processing and Real-Time Industrial System, Wuhan University of Science and Technology, Wuhan 430065, China; 3 Hubei Key Laboratory of Inland Shipping Technology, Wuhan 430063, China; whuthgz@whut.edu.cn; 4 School of Navigation, Wuhan University of Technology, Wuhan 430063, China

**Keywords:** low-light image enhancement, multi-scale gated denoiser, low-frequency illumination enhancer, frequency domain processing, color space conversion

## Abstract

This paper proposes a novel Denoiser and Low-Frequency Enhancer Network (DLFE-Net) for Low-Light Image Enhancement (LLIE). The DLFE-Net addresses two key challenges: (1) overexposure and detail loss in local areas during enhancement, and (2) the effective removal of inherent noise in low-light images. Specifically, the input RGB image is first converted to the HVI color space. The intensity (I) and color (H, V) maps are then enhanced and denoised separately, i.e., preserving details and removing noise. For preserving details, the Low-Frequency Illumination Enhancer (LFIE) module isolates and processes the image’s low-frequency information. This targeted approach effectively mitigates local overexposure and preserves fine details during enhancement. For removing noise, the Multi-Scale Gated Denoiser (MSGD) module performs denoising through strong preservation after predicting image noise. Comprehensive experiments were conducted on three benchmark datasets (LOL, SICE, Sony-Total-Dark) and five unpaired datasets. Both qualitative and quantitative analyses demonstrated the superiority of DLFE-Net over state-of-the-art methods. Moreover, ablation studies demonstrated the effectiveness of each module in DLFE-Net.

## 1. Introduction

In low-light conditions, image sensors are susceptible to noise and have a limited dynamic range, which degrades image quality. Low-light Image Enhancement (LLIE) techniques address this by improving signal-to-noise ratio and dynamic range distribution, restoring lost details for clearer images and better performance in downstream tasks like object detection [1,2] and re-identification [3,4]. While traditional methods like adaptive histogram equalization [5] and Retinex [6] are widely used, they often introduce noise and overexposure, limiting their effectiveness. These issues not only compromise enhancement quality but also negatively impact downstream task performance.

In recent years, researchers have proposed different solutions to the above problems. Some methods [7,8] combine Retinex and deep learning to improve the problem of image detail loss, but there is a drawback of noise amplification when dealing with extremely low-light scenes. Some methods [9,10] integrate degraded representations from low-light images into diffusion models, using the diffusion process to gradually remove noise from the image while gradually restoring details. However, existing diffusion model-based methods for modeling noise are still limited to the assumption of additive Gaussian noise, making it difficult to handle complex noise mixture distributions in real scenes. There are also some methods [11,12,13,14] that use attention mechanisms to focus the model on low-light parts and areas with high noise distribution in the image. Although progress has been made in noise suppression, the global attention map constructed by them makes it difficult to accurately capture local degradation features under non-uniform lighting conditions.

Recently, the HSV color space has shown significant advantages in the LLIE field due to its ability to effectively separate color and brightness information from images [15,16]. By targeting the chromaticity and brightness channels separately, the HSV space can not only achieve more accurate denoising and enhancement operations but also effectively solve the common color distortion and detail loss problems of traditional methods in low-light environments. However, in HSV space, there are issues with red discontinuous noise and black plane noise. Yan et al. [17] proposed polarizing HS into orthogonal HV (Horizontal/Vertical) components and introducing an adaptive intensity compression function $C_{k}$, which effectively eliminates red discontinuous noise and black plane noise, laying an important theoretical foundation for our research.

**Figure 1:**
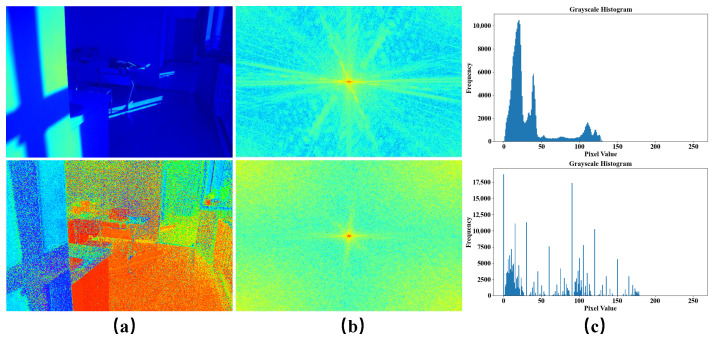
Comparison of pixel frequency distribution histograms and spectrograms between intensity and HV color maps. Among them, (**a**) represents the intensity map (above) and the HV color map (below), (**b**) represents the spectrogram and corresponds one-to-one with (**a**), and (**c**) represents the histogram of pixel value frequency distribution and corresponds one-to-one with (**a**).

We propose a Denoiser and Low-Frequency Enhancer Network (DLFE-Net) to address the difficulty in effectively removing noise in low-light images and the loss of image detail information and local exposure during the enhancement process. DLFE consists of Multi-Scale Gated Denoiser (MSGD) and Low-Frequency Illumination Enhancer (LFIE). As shown in Figure 1, in the HV color map, we can observe the high-frequency noise contained in low-light images. The intensity map mainly contains information on light distribution, which is usually manifested as low-frequency features that depend on long distances. Therefore, we use MSGD to denoise the HV color map while employing LFIE to enhance the illumination intensity map. On the one hand, the MSGD module first extracts noise features through multi-scale dilated convolution, then it fuses multi-scale features through channel attention, and it finally performs gate modulation denoising based on predicted noise maps. On the other hand, the LFIE module separates high-frequency and low-frequency information in the image, performs deep processing on low-frequency information, and weakens high-frequency information. This separation processing method can effectively improve the overall lighting distribution while preserving key texture features. The main contributions of this work are summarized as follows:

- We introduce the MSGD module in the HV branch, which first uses multi-scale dilated convolution to extract noise features and then achieves adaptive denoising through gate modulation, effectively eliminating the influence of noise in low-light images.
- We introduce the LFIE module in branch I, which first separates image information into low-frequency and high-frequency information, and then performs deep enhancement processing on the low-frequency information. This processing method effectively solves the problem of local overexposure in LLIE, while avoiding the possible loss of details during the enhancement process.
- A large number of experimental results on nine datasets demonstrate that our proposed DLFE method achieves state-of-the-art performance in LLIE tasks.

## 2. Related Work

### 2.1. Low-Light Image Enhancement

**Traditional methods.** Traditional LLIE methods, including histogram equalization [5], gamma correction [18], and Retinex methods [6], can improve image contrast but often produce unnatural results. Histogram equalization may cause some regions to be overenhanced while leaving others underenhanced. Gamma correction leads to uneven brightness distribution and loss of image details. Retinex-based approaches suffer from halo artifacts and noise amplification because their Gaussian filtering cannot accurately separate illumination variations from object edges. These conventional methods struggle with complex illumination and often amplify noise during enhancement.

**Deep learning methods.** In recent years, many deep learning-based LLIE methods have been proposed. Some methods [7,11,19,20,21,22] focus on improving image brightness. For example, Brateanu et al. [19] adopted a YUV channel separation strategy, using a CWD module for noise suppression and MHSA for illumination adjustment. Cai et al. [7] proposed a single-stage Retinex framework and an illumination-guided Transformer architecture to simultaneously handle illumination adjustment and noise suppression. However, these methods often struggle to balance denoising and enhancement tasks in complex lighting environments. Other methods [9,10,23,24,25] employ diffusion models to jointly accomplish denoising and enhancement, but their progressive recovery process leads to a significant increase in computational complexity, severely impacting the model’s inference efficiency. We decouple images into HV color maps and intensity maps based on the HVI space, enhancing the intensity map while denoising the HV color map. This enhancement strategy not only achieves specialized processing of denoising and enhancement tasks through a dual-branch network but also employs a single-iteration denoising module, significantly improving computational efficiency while ensuring performance.

### 2.2. Frequency Domain in Low-Light Image Enhancement

Recent research has found that by converting images from the spatial domain to the frequency domain and processing them in the frequency domain, this method exhibits significant performance improvements in LLIE. Liang et al. [26] performed adaptive processing on high-frequency and low-frequency information in images, improving overall brightness while enhancing texture details and edge information. Huang et al. [27] set up a dual-stream encoder to process information in both the spatial and frequency domains, thereby improving the brightness and contrast of the image. He et al. [23] used a wavelet transform to separate the high and low-frequency information of images, and performed a Fourier transform in the low-frequency domain to obtain amplitude and phase information, thus compensating for the lack of illumination and structural information. These methods all process images in the frequency domain and have achieved remarkable results, laying a solid theoretical foundation for our research.

## 3. Methods

### 3.1. Network Architecture

**Figure 2:**
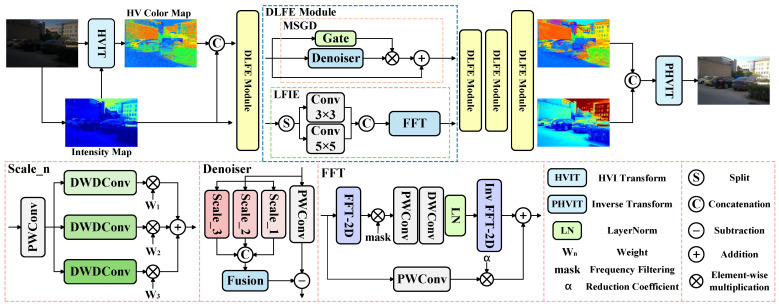
The overall network of DLFE proposed by us. MSGD is applied to the HV branch, and LFIE is applied to the I branch. The self-attention module and feedforward network after the MSGD and LFIE modules have been omitted in the figure.

As shown in Figure 2, we convert the RGB color space to the HVI color space and apply a denoising task to the HV branch and an enhancement task to the I branch. The input of the HV branch combines the intensity map and HV color map, as the brightness component of extremely low-light images also contains noise [17]. Furthermore, unlike CIDNet [17], which lacks explicit denoising and enhancement modules, our approach achieves more refined processing through two key modules: the MSGD module performs denoising on the color branch in a strong-preservation manner, while the LFIE module enables targeted enhancement by decoupling the low- and high-frequency components of the illumination. The specific details of these modules are described in more detail below.

### 3.2. Multi-Scale Gated Denoiser

Images captured in low-light environments often contain noise, which can interfere with the color information of the image. Inspired by [28], we propose a new MSGD module. The MSGD module consists of a noise estimation module and a noise modulation module. We discuss these two modules in detail below.

**Noise Estimation Module.** Unlike [28], which estimates noise in images from both local and global perspectives, we adopt a multi-scale approach to estimate noise in low-light images. A three-level hierarchical perception structure is adopted at each scale $S_{n}$. In the three-level hierarchical perception structure, we set three convolutions $Conv_{d_{i}}\left(\cdot \right)$ with different dilation rates and assign a learnable weight coefficient $W_{i}$ to each convolution. We then weigh and sum the noise features extracted from each convolution:

(1) $$S_{n}=\sum_{i=1}^{3}W_{i}\cdot Conv_{d_{i}}\left(X\right)$$

Specifically, we use [1, 2, 3] small dilation convolutions designed to capture pixel-level high-frequency noise. We configure [4, 5, 6] medium dilation convolutions at the mesoscale to focus on extracting noise patterns in local regions. At the macro scale, a large dilation convolution of [7, 8, 9] is used specifically to capture the global noise distribution characteristics of the image. Afterward, multi-scale fusion is performed through the fusion module. Among them, Concat(·) is the concatenation of noise features according to channel dimensions, CAB(·) is a lightweight channel attention module designed to further fuse multi-scale noise features and suppress unreliable noise estimations, and N is the estimated noise:

(2) $$N=CAB(PWConv\left(Concat\left(S_{1},S_{2},S_{3}\right)\right))$$

**Noise Modulation Module.** After estimating the noise distribution of low-light images, we dynamically adjust the fusion strength of denoising residuals through a gating mechanism, which is not present in [28]. Firstly, we input X into ProjConv(·) and use a learnable linear transformation to map the input signal to a feature space that is more suitable for noise separation, resulting in a cleaner image with higher separability from noise in the projected space. The specific formula is as follows, where ProjConv(·) is a point-wise convolution, and R is the residual for denoising:

(3) R=ProjConv(X)−N

However, the noise estimation module may mistakenly identify image details as noise. If the aforementioned denoising method is directly applied, it will lead to the loss of image detail information. Therefore, we introduce a gating mechanism to achieve a dynamic balance between noise removal and detail preservation:

(4) Gate(X)=Sigmoid(PWConv(X))

(5) $$X_{output}=X+Gate\left(X\right)⊙R$$

First, in regions where the noise estimation module makes incorrect judgments, reducing the gating value effectively preserves detailed image information. Second, for correctly identified noise regions, increasing the gating value enhances denoising performance while maintaining feature integrity through residual connections. More detailed information is given in ‘Comparison of Different Denoising Methods’ in Section 4.4.

### 3.3. Low-Frequency Illumination Enhancer

Image detail loss and local exposure issues may occur in LLIE tasks. Inspired by [29], we innovatively propose the LFIE module. The module decomposes the image into high- and low-frequency information, transforming the complex light optimization problem into a targeted enhancement of the low-frequency information. This approach enables the model to focus on modeling the global illumination distribution, thereby effectively avoiding local overexposure while preserving high-frequency details during the enhancement process. The LFIE module consists of a multi-scale spatial perception module and a low-frequency information enhancement module. We now discuss these two modules in detail.

**Multi-scale Spatial Perception Module.** To provide more comprehensive frequency-domain input for subsequent frequency-domain processing, we set up a multi-scale spatial perception module before the low-frequency information enhancement module. The module first decomposes the input features along the channel dimension and sends them to the 3 × 3 and 5 × 5 convolution branches for parallel processing. The 3 × 3 convolution focuses on capturing high-frequency detail components, while the 5 × 5 convolution focuses on extracting low-frequency lighting features. By concatenating features in the channel dimension, this multi-scale design ensures that the Fourier transform can synchronously process key information in different frequency bands:

(6) $$X=Concat(Conv_{multi}\left(Split\left(X\right)\right)$$

**Low-frequency Information Enhancement Module.** Unlike [29], we separate low-frequency and high-frequency information and process low-frequency information after converting images from the spatial domain to the frequency domain. Specifically, we first construct frequency-domain coordinates and convert the height and width of the image spatial domain into normalized frequency coordinates ${\left\{u_{i}\right\}}_{0}^{H-1}$ and ${\left\{v_{j}\right\}}_{0}^{W//2}$ through linear mapping. The vertical index *i* covers all spatial domain sampling points from 0 to H−1, ensuring the completeness of the frequency-domain components. The normalized coordinates ${\left\{u_{i}\right\}}_{0}^{H-1}\in \left[-1,1\right]$ are used to center the zero frequency. The horizontal index *j* covers the non-redundant frequency-domain components of the real FFT output from 0 to W//2, ensuring that the Nyquist frequency is correctly preserved at even widths. Simultaneously normalized coordinates ${\left\{v_{j}\right\}}_{0}^{W//2}\in \left[0,1\right]$:

(7) $$u_{i}=-1+\frac{2i}{H-1},\;v_{j}=\frac{j}{W//2},\;i\in \left\{0,1,\ldots ,H-1\right\},j\in \left\{0,1,\ldots ,W//2\right\}$$

Subsequently, based on the distance characteristics between the frequency coordinates $\left(u_{i},v_{j}\right)$ and the frequency center, we designed a Gaussian mask M. When the coordinate point is close to the frequency center, the mask value tends to 1 to enhance low-frequency information. To achieve adaptive control of different frequency bands, we introduce a learnable parameter β to dynamically adjust the attenuation rate of the Gaussian mask, enabling the model to automatically adjust the frequency band division based on the frequency-domain characteristics of the input image:

(8) $$M_{ij}=exp\left(-\frac{\sqrt{u_{i}^{2}+V_{j}^{2}}}{\sigma \cdot \beta }\right),\;u_{i}\in \left[-1,1\right],\;v_{j}\in \left[0,1\right]$$

Among them, σ is the base scaling factor, and β is the learnable parameter.

Finally, we perform the Fourier transform on the input feature X and multiply the obtained frequency-domain representation element by element with a Gaussian mask to extract low-frequency components. The processed low-frequency information is fused with the appropriately attenuated original features through residual connections. This design aims to effectively enhance lighting information while fully preserving the key detail features of the image:

(9) $$X_{output}=F^{-1}\left(C_{low}\left(F\left(X\right)⊙M\right)\right)+PWConv\left(X\right)⊙\alpha$$

Among them, $C_{low}$ represents a series of convolution operations, and α is the decay parameter.

### 3.4. Loss Function

In order to integrate the advantages of RGB space and HVI space, we convert the RGB space of the normal-light image to HVI space and constrain it with the output HVI image, and at the same time convert the output HVI image to RGB space and constrain it with the normal-light image. Where $\lambda_{HVI}$ is set to 1 in the experiment, and other hyperparameter settings are specified in the experimental section:

(10) $$L_{RGB}=\lambda_{L1}L_{L1}+\lambda_{ssim}L_{ssim}+\lambda_{edge}L_{edge}+\lambda_{perc}L_{perc}$$

(11) $$L_{HVI}=\lambda_{L1}L_{L1}+\lambda_{ssim}L_{ssim}+\lambda_{edge}L_{edge}+\lambda_{perc}L_{perc}$$

(12) $$L_{total}=L_{RGB}+\lambda_{HVI}L_{HVI}$$

## 4. Experiments

### 4.1. Datasets and Evaluation Metrics

We evaluate using nine commonly used LLIE benchmark datasets, including LOLv1 [30], LOLv2 [31], DICM [32], LIME [33], MEF [34], NPE [35], VV [36], SICE [37], and Sony-Total-Dark. For paired datasets, we evaluate using the Peak Signal-to-Noise Ratio (PSNR), the Structural Similarity (SSIM) [38], and the Learned Perceptual Image Patch Similarity (LPIPS) [39] evaluation metrics, while for unpaired datasets, we evaluate using the Natural Image Quality Evaluator (NIQE) [40] evaluation metrics.

**LOL.** The LOL dataset includes two main variants: LOLv1 [30] and LOLv2 [31]. In LOLv1, there are 485 paired training images along with 15 test pairs. LOLv2 is further divided into LOLv2-real and LOLv2-synthetic: the former comprises 689 training pairs and 100 test pairs of real-captured images, while the latter contains 900 synthetic training pairs and 100 synthetic test pairs. During training, we process LOLv1 and LOLv2-real by extracting 400 × 400 patches from each image, followed by model optimization over 1500 epochs using a batch size of 8. For LOLv2-synthetic, we adopt a slightly different approach, cropping images into 384 × 384 patches and training for 500 epochs with a batch size of 1.

**Unpaired Datasets.** For unpaired datasets, such as DICM [32], LIME [33], MEF [34], NPE [35], VV [36], we test the model trained on the LOLv2-synthetic dataset to evaluate its generalization ability.

**SICE.** The SICE dataset [37] includes 4803 training images captured in varying lighting conditions, including low-light and overexposed scenarios. For testing, we employ two subsets SICE-Mix and SICE-Grad [41] totaling 589 evaluation images. During training, images are divided into 160 × 160 patches, and the model is optimized over 1000 epochs using a batch size of 10.

**Sony-Total-Dark.** This dataset was modified and constructed based on a subset of the SID [42], where the training set contains 1866 images captured by sensors under extremely low-light conditions, and the test set consists of 598 images captured at different exposure times, covering various lighting conditions from short-exposure low-light to long-exposure normal-light. To increase the complexity of the task, we processed the raw images by converting them to sRGB format without applying gamma correction, producing significantly darker outputs. We cropped the training images into 256 × 256 patches and trained the model for 1000 epochs with a batch size of 4.

### 4.2. Implementation Details

**Figure 3:**
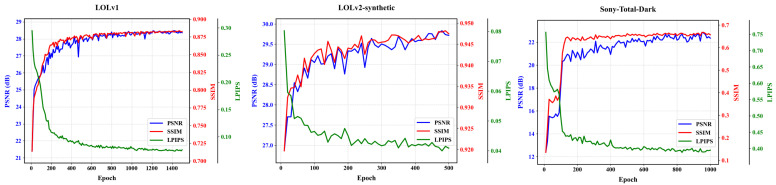
Model performance curve with the number of training rounds.

We trained our U-net-based [43] DLFE-Net using a single RTX 4090 GPU. In terms of the optimizer, we chose the Adam optimizer [44] and set the learning rate to $1\times 10^{-4}$. Then, during the training process, we gradually reduced it to $1\times 10^{-7}$ through a cosine annealing scheme [45]. In terms of weight setting for the loss function, for the LOLv1 and LOLv2-synthetic datasets, we set weights of 1.00, 200.00, and 0.01 for SSIM loss [38], edge loss [46], and perceptual loss [47], respectively. For the LOLv2-real and Sony-Total-Dark datasets, we only set weights of 0.01 for perceptual loss. For the SICE dataset, we set weights of 50.00 and 0.01 for edge loss and perceptual loss, respectively. In addition, we used gamma correction to train the model on the SICE dataset. As shown in Figure 3, we provide the variation of model performance with the number of training rounds on the LOLv1, LOLv2-synthetic, and Sony-Total-Dark datasets.

### 4.3. Comparisons with States of the Art

**Figure 4:**
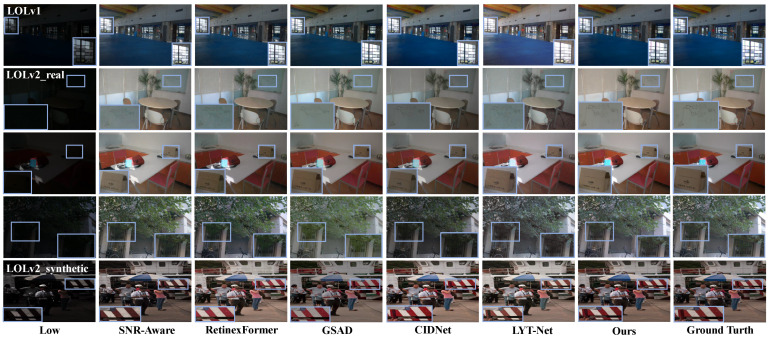
Visual comparisons of enhancement results by using different methods: SNR-Aware [11], RetinexFormer [7], GSAD [10], CIDNet [17], and LYT-Net [19] on the LOLv1, LOLv2-real, and LOLv2-synthetic datasets.

**Results on LOL Datasets.** As shown in Table 1, our method achieves optimal results in PSNR and LPIPS metrics. This is because the MSGD module effectively suppresses noise in low-light images, significantly reducing pixel-level errors and achieving optimal PSNR performance. At the same time, the LFIE module focuses on low-frequency information processing, avoiding local overexposure issues during image enhancement, and achieving lighting adjustments that are more in line with human perception. Therefore, the LPIPS performance is optimal. As shown in Figure 4, in the first line, our method targets low-light images with uneven lighting distribution, effectively avoiding the problem of local overexposure during the enhancement process and solving the problem of detail loss during image enhancement. In the second line, compared to other methods, our approach not only effectively suppresses noise but also better preserves key details in the image. In the third line, facing the interference of high-brightness areas in low-light images, our method avoids the overall brightness decrease problem that occurs with other methods. In the fourth line, our method successfully solves the problem of overexposure or insufficient enhancement that other methods have in local areas. In the fifth line, compared to the color distortion caused by other methods, our method maintains accurate color reproduction when enhancing the detailed areas of the image.

**Results on SICE and Sony-Total-Dark.** To validate the performance of our model under extreme low-light conditions and mixed low-light and exposure conditions, we tested the model on the SICE and Sony-Total-Dark datasets. As shown in Table 2, the test results on the SICE dataset indicate that our model can better adapt to different lighting distributions, owing to the LFIE module’s focused processing of low-frequency information in images. In addition, each image in the SICE-Mix and SICE-Grad datasets is mixed with three conditions: low-light, normal-light, and exposure. The test results of the model on these two datasets further demonstrate its excellent generalization ability and adaptability to extreme conditions. The test results on the Sony Total Dark dataset show that our model not only performs best under extreme low-light conditions but also effectively suppresses the noise generated by the sensor in extreme low-light environments.

**Figure 5:**
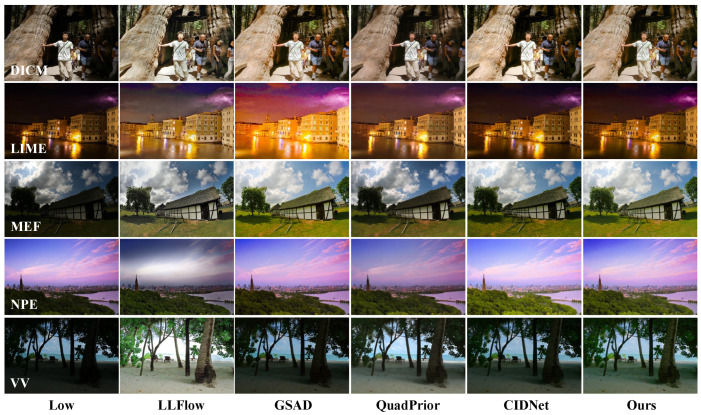
Visual comparisons of enhancement results by using different methods: LLFlow [52], GSAD [10], QuadPrior [25], and CIDNet [17] on the DICM, LIME, MEF, NPE, and VV datasets.

**Results on Unpaired Datasets.** We use the NIQE evaluation metric [40] to assess the visual quality of unpaired images after enhancement. As shown in Table 3, our method achieves optimal performance on the DICM, LIME, and MEF datasets, and suboptimal performance on the NPE datasets. This indicates that our enhancement results have advantages in naturalness and visual realism, avoiding the problem of local exposure in images caused by excessive processing. As shown in Figure 5, our method exhibits significant advantages in low-light image enhancement, especially when dealing with images with uneven lighting distribution. In the first four lines of the example, our method avoids the problem of local overexposure during low-light image enhancement. For the fifth line, compared with the LLFlow method, our method enhances the image while fully preserving the cloud details in the background, while the comparative method shows a significant loss of details.

### 4.4. Ablation Studies

**Figure 6:**
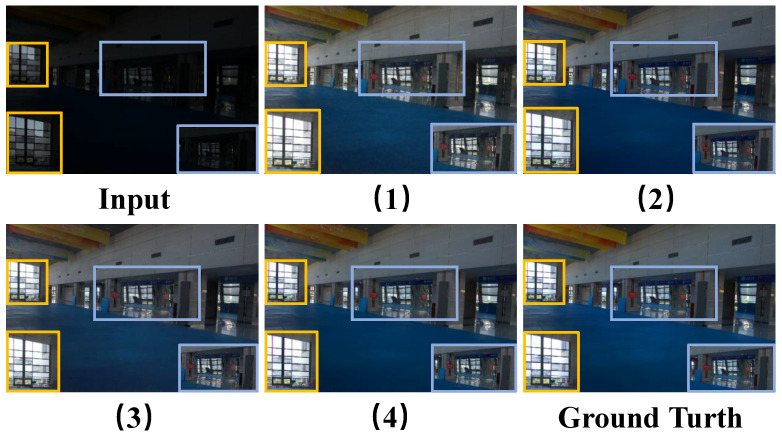
Visual comparisons of enhancement results by using four ablation experiments on the LOLv1 dataset. Among them, (1), (2), (3), and (4) correspond one-to-one with Table 4.

**Comparison between Various Modules.** We conducted four quantitative ablation experiments on the LOLv1 dataset to validate the feasibility of each module. As shown in Table 4, firstly, Experiment 2 and Experiment 3 are higher than Experiment 1 in all evaluation indicators, which directly reflects the feasibility of each module. Secondly, Experiment 2 achieves significant improvements in PSNR and SSIM metrics compared to Experiment 1, mainly due to LFIE’s focus on processing the low-frequency components of intensity maps. By optimizing and adjusting low-frequency information, the model can effectively improve the overall brightness distribution of the image, allowing darker areas to receive more enhancement while brighter areas remain relatively stable. This processing method makes the enhanced image closer to the real image in terms of brightness distribution, thereby directly reducing the pixel difference between the two, which is the main reason for the improvement of PSNR. At the same time, due to the improvement of brightness distribution, the similarity of the image structure is also enhanced, so the SSIM index is correspondingly improved. Finally, Experiment 3 achieves a more significant improvement in PSNR compared to Experiment 2. This is because MSGD directly acts on the HV color map, which can more accurately estimate the noise in the image and, thus, perform targeted denoising. As shown in Figure 6, we compare an image on the LOLv1 test set. Experiment 2 achieves better restoration of details outside the left window compared to Experiments 1 and 3 under the lighting optimization effect of the LFIE module. At the same time, Experiment 3 shows superior noise suppression performance in the middle area of the image, with its noise reduction performance significantly surpassing that of Experiments 1 and 2. In addition, as shown in Table 5, we tested the computational complexity of each module to provide more detailed information about each module.

**Feasibility Study Focusing on Low-Frequency Processing.** In the intensity map, the information of light distribution mainly exists in the low-frequency components. Based on this characteristic, we adopted a frequency-domain separation method to decompose the image into high- and low-frequency components and process the low-frequency information in a targeted manner to optimize the global illumination distribution and solve the problem of local overexposure during the enhancement process. To verify the above theory, we conducted four quantitative experiments. The first experiment extracted high-frequency information for independent processing, the second experiment fully segregated both high and low frequencies and processed them separately, the third experiment processed high and low frequencies jointly without any separation, and the fourth experiment isolated low-frequency information for dedicated processing. As shown in Table 6, the low-frequency emphasis method performs the best in all evaluation indicators. This method achieves dual advantages by separating low-frequency information and processing it specifically to adjust illumination uniformity, while using a reduction coefficient to control the contribution of original features. It effectively addresses the issue of local overexposure while avoiding the loss of high-frequency information and the amplification of noise. In contrast, the high-frequency emphasis method has obvious shortcomings. Because the high-frequency region in the illumination intensity map contains fewer effective details and noise [17], enhancing the high-frequency component will significantly amplify the noise and cannot improve the problem of uneven low-frequency illumination. Although the high- and low-frequency complete separation method performs well in some indicators, it significantly deteriorates the LPIPS index due to the destruction of the natural correlation between frequency bands, reflecting a decrease in perceptual quality. Although the method of not separating high and low frequencies is superior to the high-frequency focused method, due to the lack of band-specific processing, it cannot differentiate and optimize low-frequency lighting and high-frequency details, and it still enhances high-frequency noise. The overall comparison shows that the low-frequency emphasis method exhibits the most comprehensive and optimal performance due to its targeted frequency band processing strategy.

**Figure 7:**
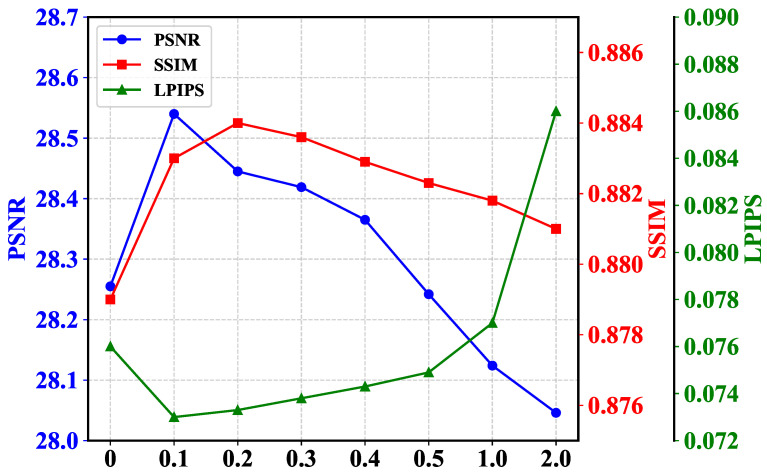
Comparison of quantitative test results for different residual contribution rates on the LOLv1 dataset. The horizontal axis represents the residual ratio, and the vertical axis represents the size of the test indicators.

**Residual Contribution Ratio.** As shown in Figure 2, in the LFIE module, we set a reduction coefficient to control the contribution of the residual part. By changing this value, the model can achieve different effects. As shown in Figure 7, as the residual contribution ratio increases, the values of all evaluation indicators first become better and then worse. This is because the residual part contains a lot of high-frequency information, which includes texture features, edge details, and noise in the intensity map [17]. If the residual is appropriately introduced, it can effectively compensate for the loss of image details after low-frequency separation. However, if the residual is excessively introduced, on the one hand, it will amplify the noise of the image, and on the other hand, it will suppress the processed low-frequency information with high-frequency information. At the same time, if the residual part is completely discarded, it is equivalent to losing some of the detailed information of the image, and the evaluation index will also significantly decrease. After considering all evaluation indicators comprehensively, we chose 0.1 as the reduction coefficient.

**Comparison of Different Denoising Methods.** We use a gate-controlled weighting mechanism to process the denoised features and then perform residual connections with the original features, aiming to effectively preserve the detailed information of the image during the denoising process. To verify the effectiveness of this method, we designed three sets of quantitative experiments. The first group weights the estimated noise through a gating weighting mechanism and directly denoises it, which is called direct denoising. The second group balances the original features and denoised features through a gating weighting mechanism, which is called weak preservation denoising. The third group uses a gate-weighted mechanism to process the denoised features, which is called strong preservation denoising. The following shows the specific formulas for three denoising methods, and the group numbers after the formulas correspond one-to-one with the group numbers in Table 7:

(13) $$X_{output}=\left\{\begin{array}{cc} X-Gate(X)⊙N, & Group\;(1)\\ (1-Gate(X))⊙X+Gate(X)⊙(ProjConv(X)-N), & \;Group\;(2)\\ X+Gate(X)⊙(ProjConv(X)-N), & \;Group\;(3) \end{array}\right.$$

As shown in Table 7, the third group of experiments achieved the best performance in terms of the four evaluation indicators. For direct denoising, subtracting the weighted noise from the original features can effectively suppress noise, but it will excessively smooth the image, resulting in texture and edge loss. Compared to direct denoising, weak preservation denoising may seem to compromise denoising and detail preservation. However, if the gating value is small, the original features are amplified while the noise is not sufficiently suppressed. If the gating value is large, this method is equivalent to direct denoising, which will result in the loss of details. Finally, for strong preservation denoising, we model the original features as $X=X_{clean}+N$. When the gate value approaches 1, it indicates that the estimated noise is approaching the true noise. Therefore, the denoising formula becomes

(14) $$X_{output}=2X_{clean}+N$$

**Figure 8:**
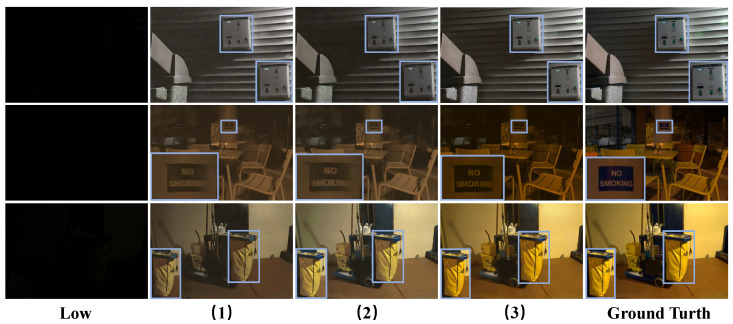
Visual comparisons of enhancement results by using different denoising methods on the Sony-Total-Dark dataset. Among them, (1), (2), and (3) correspond one-to-one with Table 7.

Although residual connections are used to introduce noise from the original features, the clean image portion is doubled in size. Through the layer-by-layer denoising of the UNet network’s encoder and decoder, the clean portion can be gradually enlarged while gradually reducing noise, achieving the goal of denoising while preserving image details. As shown in Figure 8, under extremely low-light conditions, both direct denoising and weak preservation denoising methods will smooth out the detailed features of the image to some extent, leading to color distortion problems. In contrast, strong preservation denoising methods not only effectively denoise, but also better preserve image details and features. Therefore, we choose this method to denoise the HV color map branches.

**Figure 9:**
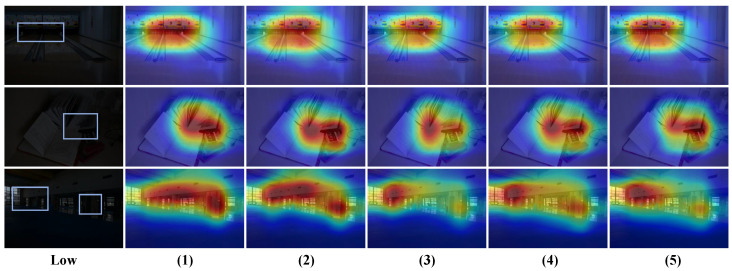
Comparison of heatmaps with different dilation rates on the LOLv1 dataset. Among them, (1), (2), (3), (4), and (5) correspond one-to-one with Table 8. In addition, the areas marked with boxes in low-light images are the key areas that the model should focus on.

**Comparison of different dilation rates.** In the noise estimation module, we adopt a multi-scale approach with a three-level hierarchical perception structure at each scale to estimate noise in low-light images. We test various dilation rate combinations to evaluate their impact on model performance. As shown in Table 8, the progressive dilation rate combination demonstrates the best performance, which benefits from its ability to capture local details hierarchically, mid-range correlations, and globally distributed noise patterns, thereby achieving comprehensive noise modeling. In contrast, single global or local dilation rates each have significant drawbacks: global captures large-scale noise distributions but misses local details, while local preserves high-frequency information but fails to capture global structures. This confirms the necessity of multi-scale feature fusion in noise estimation. Additionally, although the skip dilation rate combination attempts to expand the receptive field range, the lack of continuity between dilation rates disrupts the correlation between local and global noise patterns, adversely affecting feature fusion. Without dilated convolutions, this case preserves high-frequency noise better but only captures pixel-level features, lacking contextual image information for accurate noise estimation. As shown in Figure 9, the heatmap analysis provides more intuitive insights into the performance differences among various dilation rate combinations. For the global dilation rate combination, the heatmap shows that the model applies uniform attention across the image, failing to distinguish intensity variations in noisy regions, which hinders accurate identification of critical noise areas. The large gaps between dilation rates in the skip dilation combination weaken the correlation between local and global noise patterns, causing the model’s focus to deviate from actual noise distributions. As for the case without dilation rates, the heatmap shows that its attention scope is overly limited, capturing only pixel-level local noise features. Finally, while both local and progressive dilation rate combinations effectively focus on noise-dense regions, the latter exhibits clearer multi-scale attention in the heatmap: small dilation rates capture fine details, medium ones cover regional patterns, and large ones establish global correlations. This hierarchical attention pattern aligns well with the actual distribution characteristics of noise, leading us to ultimately select the progressive dilation rate strategy as the optimal solution.

**Figure 10:**
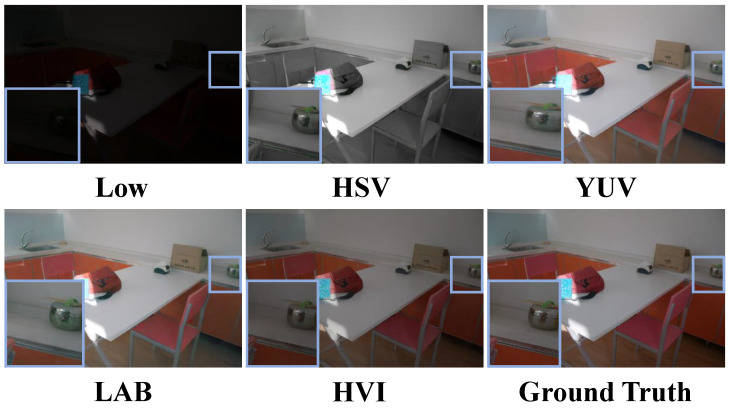
Visualization results for different color spaces on the LOLv2-real dataset. The HSV and LAB color spaces show color deviation problems marked with blue boxes.

**Comparison of different color spaces.** In order to justify the choice of HVI color space, we performed multiple color space transformations on the RGB images and decoupled them into illumination components and color components, which were processed using the same network architecture. As shown in Table 9, although the LAB color space is designed to take into account the perceptual characteristics of the human eye on color change, so that its color difference calculation is more in line with human visual perception, the a-axis and b-axis obtained from its decoupling have the problem of difficult color adjustment during model processing, especially in the low-illumination region that is prone to the color bias phenomenon. While the YUV color space was originally designed for video transmission, the chromaticity downsampling technique it adopts effectively reduces the amount of data but inevitably causes the loss of color information. As shown in Figure 10, in the HSV color space, the calculation of the Saturation depends on the Value. When the luminance decreases, the maximum effective saturation of the color will show a nonlinear decay characteristic, which leads to color distortion. Through the comprehensive analysis above, compared to other color spaces, HVI shows superior performance.

### 4.5. Inference Time on Device

To comprehensively evaluate the model’s practical deployment performance, we conducted systematic testing on both the 13th Gen Intel(R) Core(TM) i7-13650HX CPU and RTX 4060 GPU platforms. Our evaluation focused on two key metrics: CPU/GPU utilization and inference latency. As shown in Table 10, we performed repeated experiments across multiple test sets from different datasets, collecting multiple measurements to calculate mean values and standard deviations, thereby ensuring the reliability and statistical significance of our experimental results.

### 4.6. Analysis of Failure Cases

**Figure 11:**
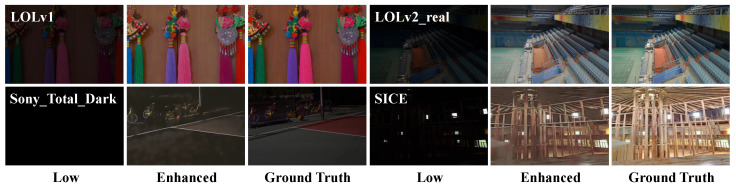
Comparison chart of failure case results. Among them, from left to right are the low-light image, the enhanced image, and the label image in that order.

In order to fully evaluate the performance of DLFE-Net, we provide some failure cases and analyze them. As shown in Figure 11, our method exhibits issues of localized detail blurring and color deviation when processing extremely low-light regions in images. Specifically, when dealing with extremely low-light areas where brightness is nearly completely absent, although the noise modulation module in MSGD can preserve some image detail information during denoising, the degradation in these regions is so severe that the model struggles to extract authentic image features. This makes it difficult for the model to accurately distinguish between noise and structural image information, leading to excessive denoising in these areas and ultimately resulting in edge smoothing. Regarding color deviation, this issue stems from the inherent limitations of the HSV and HVI color spaces. In the HSV space, the Value component is calculated based on the maximum RGB theory. However, noise interference in low-light images can excessively amplify the Value component, thereby widening the gap between the maximum and minimum channel values. This not only weakens the chrominance signal but also affects the accurate calculation of the Saturation, ultimately leading to color distortion. More critically, excessive noise interference can even alter the relative magnitudes of RGB channel values, causing a fundamental shift in the dominant hue. This phenomenon is particularly evident in the Sony-Total-Dark dataset. Since the H and V components in the HVI space are calculated from the Hue and Saturation in HSV, the aforementioned issue exists not only in HSV but also in HVI.

## 5. Conclusions

This paper proposes DLFE-Net, a novel network addressing critical challenges in low-light image enhancement (LLIE): local overexposure with detail loss and persistent noise suppression. Specifically, DLFE-Net contains two modules: Low-Frequency Illumination Enhancement (LFIE) and Multi-Scale Gated Denoising (MSGD) modules. The RGB images are transformed into the HVI color space, enabling independent enhancement and denoising of the intensity (I) and color (H, V) components. The LFIE module selectively processes low-frequency illumination information, effectively mitigating localized over-enhancement and preserving intricate details. The MSGD module adopts noise estimation and strong preservation denoising methods to preserve high-frequency details of the image during the denoising process. Extensive evaluations on diverse datasets (LOL, SICE-Mix, SICE-Grad, Sony-Total-Dark, and five unpaired datasets) demonstrate DLFE-Net’s superior performance and robust generalization: (i) LFIE excels in optimizing illumination distribution, particularly under mixed lighting conditions (validated on SICE-Mix/SICE-Grad); (ii) MSGD exhibits exceptional sensor noise suppression capability, even in extreme low-light environments (validated on Sony-Total-Dark). While DLFE-Net achieves significant performance gains, it incurs higher computational complexity. Future work will focus on developing a content-adaptive frequency processing mechanism to dynamically allocate computational resources. This aims to substantially improve inference speed while preserving the model’s performance advantages, thereby enhancing its practical application scenarios.

## Figures and Tables

**Table 1:** Quantitative comparison of different methods on the LOLv1 and LOLv2 datasets. The best and second-best results are highlighted in red and blue, respectively. “↑” (or “↓”) means that the larger (or smaller) the better. The FLOPs was tested on a single 256 × 256 image. Please note that we obtained these results from corresponding papers or by running pre-trained models published by the authors.

Methods	LOLv1			LOLv2-Real			LOLv2-Synthetic			Param (M)	FLOPs (G)
	PSNR ↑	SSIM ↑	LPIPS ↓	PSNR ↑	SSIM ↑	LPIPS ↓	PSNR ↑	SSIM ↑	LPIPS ↓		
Zero-DCE [48] _CVPR’20_	14.861	0.562	0.335	18.059	0.580	0.313	-	-	-	0.075	4.83
EnlightenGAN [49] _TIP’21_	17.483	0.652	0.322	18.640	0.677	0.309	16.570	0.734	-	8.64	61.01
DRBN [50] _CVPR’20_	19.860	0.834	0.155	20.130	0.830	0.147	23.220	0.927	-	0.58	37.79
MIRNet [51] _TPAMI’22_	24.140	0.842	0.131	20.357	0.782	0.317	21.940	0.846	-	5.90	22.75
LLFlow [52] _AAAI’22_	25.132	0.872	0.117	26.200	0.888	0.137	24.807	0.919	0.067	17.42	358.4
DDNet [53] _TITS’24_	25.530	0.823	0.182	28.374	0.861	0.165	27.938	0.927	0.069	-	-
ExpoMamba [54] _ICME’24_	25.770	0.860	0.212	28.040	0.885	0.203	-	-	-	-	-
LLFormer [55] _AAAI’23_	25.758	0.823	0.167	26.197	0.819	0.209	28.006	0.927	0.061	24.55	22.52
RetinexMamba [8] _ICONIP’25_	26.343	0.838	0.136	27.765	0.864	0.169	29.400	0.942	0.055	4.59	42.82
LYT-Net [19] _SPL’25_	26.572	0.833	0.129	28.352	0.877	0.128	26.305	0.913	0.095	0.045	3.49
SNR-Aware [11] _CVPR’22_	26.716	0.851	0.152	27.209	0.871	0.157	27.787	0.941	0.054	4.01	26.35
ECAFormer [13] _arXiv’24_	26.397	0.863	0.088	28.351	0.881	0.094	29.314	0.939	0.057	2.50	-
Diff-Retinex [24] _ICCV’23_	27.091	0.873	0.087	25.049	0.859	0.152	25.127	0.911	0.087	-	-
RetinexFormer [7] _ICCV’23_	27.167	0.848	0.125	27.667	0.855	0.165	28.965	0.937	0.059	1.53	15.85
GSAD [10] _NeurIPS’24_	27.640	0.875	0.091	28.650	0.893	0.096	28.325	0.943	0.049	17.36	442.02
CIDNet [17] _CVPR’25_	28.141	0.889	0.099	28.139	0.892	0.101	29.566	0.950	0.044	1.88	7.57
Ours	28.545	0.883	0.073	28.971	0.888	0.092	29.833	0.948	0.040	3.25	10.19
Standard Deviation	2.595	0.045	0.026	3.185	0.042	0.047	4.124	0.038	0.027	-	-

**Table 2:** Quantitative comparison of different methods on SICE and Sony-Total-Dark datasets. The best and second-best results are highlighted in red and blue, respectively. “↑” (or “↓”) means that the larger (or smaller) the better. Please note that we obtained these results from corresponding papers or by running pre-trained models published by the authors.

Methods	SICE-Grad			SICE-Mix			Sony-Total-Dark		
	PSNR ↑	SSIM ↑	LPIPS ↓	PSNR ↑	SSIM ↑	LPIPS ↓	PSNR ↑	SSIM ↑	LPIPS ↓
RetinexNet [30] _BMVC’18_	12.450	0.619	0.364	12.397	0.606	0.407	15.695	0.395	-
Zero-DCE [48] _CVPR’20_	12.475	0.644	0.334	12.428	0.633	0.382	14.087	0.090	0.813
URetinexNet [56] _CVPR’22_	10.894	0.610	0.356	10.903	0.600	0.402	15.519	0.323	0.599
RUAS [57] _CVPR’21_	8.628	0.494	0.499	8.684	0.493	0.525	12.622	0.081	0.920
LLFlow [52] _AAAI’22_	12.737	0.617	0.388	12.737	0.617	0.388	16.226	0.367	0.619
LEDNet [58] _ECCV’22_	12.551	0.576	0.383	12.668	0.579	0.412	20.830	0.648	0.471
CIDNet [17] _CVPR’25_	13.446	0.648	0.318	13.425	0.636	0.362	22.904	0.676	0.411
Ours	12.924	0.664	0.291	12.844	0.651	0.339	22.943	0.665	0.387
Standard Deviation	1.567	0.172	0.093	1.890	0.166	0.100	1.452	0.168	0.099

**Table 3:** Quantitative comparison of different methods on DICM, LIME, MEF, NPE, and VV datasets. The best and second-best results are highlighted in red and blue, respectively. Please note that we obtained these results from corresponding papers or by running pre-trained models published by the authors.

Methods	DICM	LIME	MEF	NPE	VV
Zero-DCE [48] _CVPR’20_	4.58	5.82	4.93	4.53	4.81
RUAS [57] _CVPR’21_	5.21	4.26	3.83	5.53	4.29
QuadPrior [25] _CVPR’24_	4.82	5.22	5.11	4.78	3.99
LLFlow [52] _AAAI’22_	4.06	4.59	4.70	4.67	4.04
SNR-Aware [11] _CVPR’22_	4.71	5.74	4.18	4.32	9.87
PairLIE [59] _CVPR’23_	4.03	4.58	4.06	4.18	3.57
GSAD [10] _NeurIPS’24_	4.03	4.21	3.67	4.17	3.11
RetinexFormer [7] _ICCV’23_	3.85	4.31	4.23	3.76	3.09
EnlightenGAN [49] _TIP’21_	3.57	3.67	4.23	4.07	2.69
CIDNet [17] _CVPR’25_	3.79	4.13	3.56	3.74	3.21
Ours	3.55 ± 1.28	3.75 ± 1.39	3.29 ± 0.93	3.91 ± 0.89	3.22 ± 1.28

**Table 4:** Quantitative assessment results for (a) MSGD and (b) LFIE on the LOLv1 dataset. The assessment metrics used in the experiment are PSNR, SSIM, and LPIPS. “↑” (or “↓”) means that the larger (or smaller) the better. ✓ and × represent whether the module is used or not. The best results are highlighted in red.

	(a)	(b)	PSNR ↑	SSIM ↑	LPIPS ↓
(1)	×	×	27.196	0.856	0.079
(2)	×	✓	28.185	0.880	0.075
(3)	✓	×	28.406	0.882	0.074
(4)	✓	✓	28.545	0.883	0.073

**Table 5:** The computational complexity of different modules. We tested the MSGD, LFIE, and DLFE modules on a single RTX 4090 GPU and recorded the FLOPs, parameters, and inference time on the GPU. Among them, Resolutions represents the shape of the image we used for testing.

Module	FLOPs (G)	Param (M)	GPU Runtime (s)	Resolutions (H × W × C)
MSGD	2.78	0.04	1.18	256 × 256 × 72
LFIE	4.11	0.09	0.97	256 × 256 × 72
DLFE	6.89	0.13	1.61	256 × 256 × 72

**Table 6:** Quantitative evaluation results of different frequency treatments on the LOLv1 dataset. Among them, (1) high-frequency emphasis, (2) high- and low-frequency complete separation, (3) no frequency separation, and (4) low-frequency emphasis. “↑” (or “↓”) means that the larger (or smaller) the better. The best results are highlighted in red.

Group	PSNR ↑	SSIM ↑	LPIPS ↓	RMSE ↓
(1)	27.910	0.880	0.077	10.623
(2)	28.005	0.880	0.080	10.448
(3)	28.130	0.881	0.075	10.423
(4)	28.545	0.883	0.073	9.972

**Table 7:** Quantitative evaluation results of different denoising methods on different evaluation indicators. (1) Direct denoising method, (2) weak preservation denoising method, (3) strong preservation denoising method. “↑” (or “↓”) means that the larger (or smaller) the better. The best results are highlighted in red.

Group Number	LOLv1			Sony-Total-Dark		
	PSNR ↑	SSIM ↑	LPIPS ↓	PSNR ↑	SSIM ↑	LPIPS ↓
(1)	27.683	0.879	0.083	21.116	0.636	0.417
(2)	28.010	0.880	0.081	21.672	0.647	0.410
(3)	28.545	0.883	0.073	22.943	0.665	0.387

**Table 8:** Quantitative evaluation results of different expansion rates on the LOLv1 dataset. Among them, Scale_*n*_ represents the combination of dilation rates contained in each scale. “↑” (or “↓”) means that the larger (or smaller) the better. The best results are highlighted in red.

Group Number	Scale_1_	Scale_2_	Scale_3_	PSNR ↑	SSIM ↑	LPIPS ↓
(1)	[7, 8, 9]	[7, 8, 9]	[7, 8, 9]	27.821	0.881	0.079
(2)	[1, 2, 3]	[4, 6, 8]	[9, 11, 13]	27.941	0.881	0.079
(3)	[1, 1, 1]	[1, 1, 1]	[1, 1, 1]	28.081	0.882	0.080
(4)	[1, 2, 3]	[1, 2, 3]	[1, 2, 3]	28.324	0.882	0.076
(5)	[1, 2, 3]	[4, 5, 6]	[7, 8, 9]	28.545	0.883	0.073

**Table 9:** Quantitative test results for different color spaces on the LOLv2-real dataset. Here, the RGB images were converted to different color spaces and decoupled into luminance and color components, and processed using the same network. “↑” (or “↓”) means that the larger (or smaller) the better. The best results are highlighted in red.

Color Space	LOLv2-Real		
	PSNR ↑	SSIM ↑	LPIPS ↓
HSV	25.593	0.867	0.158
YUV	27.811	0.873	0.111
LAB	27.844	0.886	0.107
HVI	28.971	0.888	0.092

**Table 10:** The results of the inference performance of the model on different datasets. Among them, the first row shows the number of images in the test set of each dataset, and the second row indicates the image resolution. The CPU/GPU utilization rate is measured multiple times to obtain the mean and standard deviation, while the inference time is measured multiple times to obtain the average value.

Datasets	LOLv1	LOLv2-Real	LOLv2-Synthetic	SICE	Sony-Total-Dark
**#Test**	15	100	100	589	598
**Resolutions (H × W)**	400 × 600	400 × 600	384 × 384	600 × 900	1424 × 2128
**CPU Utilization**	23.3 ± 1.7	22.3 ± 3.9	18.1 ± 2.4	30.1 ± 3.1	51.5 ± 3.2
**CPU Runtime (s)**	35.9	242.2	154.2	2499.9	15,927.3
**GPU Utilization**	8.9 ± 1.0	9.8 ± 1.4	7.6 ± 2.3	8.6 ± 1.8	11.9 ± 2.7
**GPU Runtime (s)**	5.9	29.4	14.1	291.8	2181.9

## Data Availability

The LOLv1 dataset can be found using this link: https://daooshee.github.io/BMVC2018website/. The LOLv2 dataset can be accessed through the following link: https://github.com/flyywh/CVPR-2020-Semi-Low-Light. The DICM, LIME, MEF, NPE, and VV datasets can be accessed through the following link: https://1drv.ms/f/s!AoPRJmiD24UphBNGBbsDmSwppNPf?e=2yGImv. The SICE dataset can be accessed through the following link: https://1drv.ms/u/s!AoPRJmiD24UphAlaTIekdMLwLZnA?e=WxrfOa. The Sony-Total-Dark dataset can be accessed through the following link: https://1drv.ms/u/s!AoPRJmiD24UphAie9l0DuMN20PB7?e=Zc5DcA. The passwords above are all “yixu”.
